# Acute Hiatal Hernia After Sleeve Gastrectomy: A Case Report

**DOI:** 10.7759/cureus.69260

**Published:** 2024-09-12

**Authors:** Ariel A Ortiz, Victor Daniel Cárdenas-Salas, Arturo Martinez Gamboa, Daniel E Moreno, Milton Alberto Muñoz Leija

**Affiliations:** 1 Bariatric Surgery, International Institute of Metabolic Medicine, Tijuana, MEX; 2 General Surgery, Universidad de Monterrey, San Nicolas de los Garza, MEX

**Keywords:** bariatric and metabolic surgery, bariatric surgery complications, gastric sleeve complications, gastric sleeve surgery, omentopexy

## Abstract

Obesity is considered the pandemic of this century. With the popularization of bariatric surgery due to its effectiveness, the number of procedures has significantly increased. One of the most performed surgeries is sleeve gastrectomy (SG). It is a safe procedure that rarely presents complications, with the most common being bleeding or staple line leaks. However, there are other less common complications or those that have been infrequently reported in the literature, such as acute hiatal hernia (AHH), which may require additional surgical intervention if it occurs. As the number of bariatric procedures performed worldwide increases, the likelihood of encountering these low-incidence complications also rises. The few cases reported in the literature describe different surgical techniques for this complication. The objective of this study is to describe the case of a female patient who developed AHH after undergoing SG, which was successfully treated with reduction and omentopexy in Mexico.

## Introduction

Sleeve gastrectomy (SG) is a commonly performed bariatric procedure for treating morbid obesity. It has a restrictive effect and hormonal action by reducing the levels of ghrelin, the appetite-stimulating hormone, in the plasma [1].

Generally, it is considered a safe procedure with a low rate of complications. The main early complications occur within the first 30 days postoperatively. These include hemorrhage, staple line leaks, intra-abdominal abscess formation, or wound infections [2]. However, there are other less common complications, such as acute hiatal hernia (AHH), which can be fatal for the patient and require timely and rapid diagnosis and treatment. Only a few cases have been reported in the literature [3-5].

With the increase in medical tourism in our region [6], bariatric surgeons must be aware of these low-incidence complications. This article reports a rare case of AHH in a female patient successfully treated in Mexico. This case report has been documented in line with the Surgical Case Report (SCARE) guidelines [7].

## Case presentation

A 44-year-old female patient with a BMI of 38.5 and no significant medical history presented to our clinic for a bariatric procedure. The patient denied any data related to gastroesophageal reflux disease or gastrointestinal discomfort. Preoperative studies and preparation were conducted according to our institutional protocols, and a laparoscopic vertical gastrectomy was decided upon.

The surgical procedure was performed under general anesthesia. The patient was placed in the French position, and a transverse periumbilical incision approach was made in a line to the left sternal area of the abdomen. The incision measured 12 mm and was carried through the layers to the abdominal cavity. Under direct vision, pneumoperitoneum was initiated with CO_2_ at 12 mmHg. Using direct laparoscopic vision, a 12-mm trocar was placed 6 cm away from the right zone of the first trocar, and two 5-mm trocars were placed in the left and right flanks at the level of the initial trocar. A Nathanson hepatic retractor was inserted subxiphoid under direct vision. No evidence of a hiatal hernia was observed upon deliberate examination of the area. The greater curvature of the stomach was released approximately 3 cm from the pylorus. Using an ultrasonic energy device, it was released up to the gastroesophageal junction, with hemostasis of the short vessels. A 32-Fr gastric calibration tube was passed toward the pylorus. A Medtronic (Dublin, Ireland) surgical stapler was used under direct vision. The staple line was reinforced with a non-absorbable suture. The cavity was irrigated with saline solution until the gastric sleeve was completely submerged. The stomach adjacent to the pylorus was occluded with a laparoscopic Babcock clamp, and no air leakage was observed from the staple line or any other site. The anterior aponeurosis in the initial wound was closed with a simple 0 polypropylene suture. The skin was closed with a 4-0 poliglecaprone subcuticular suture and 2-octyl cyanoacrylate adhesive. No drainage was placed.

**Figure 1:**
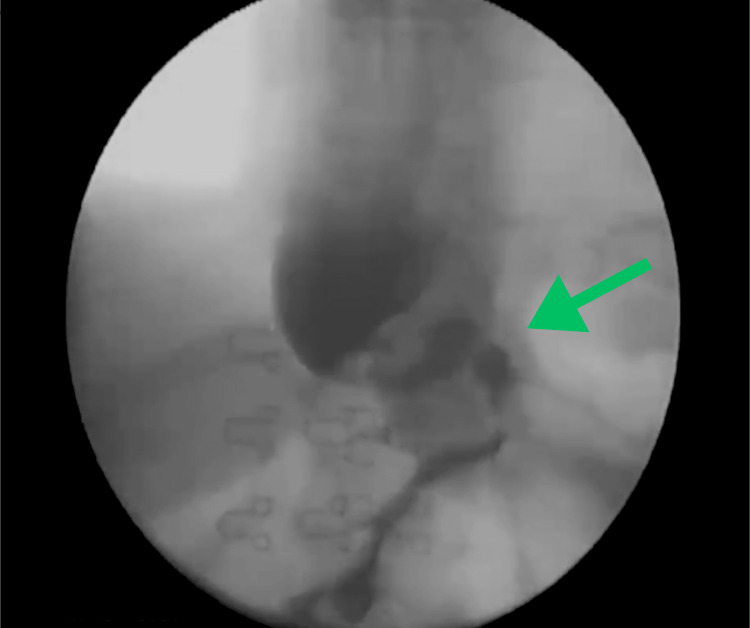
The barium swallow test after the first surgery revealed a hiatal hernia and stenosis area (green arrow).

The patient was discharged on postoperative day one after a thorough evaluation by the attending physicians, with adequate tolerance to liquids and good pain management. However, she began experiencing epigastric discomfort, frequent vomiting, and oral intolerance within 10 hours of her discharge. Upon evaluation, a barium swallow test revealed a sleeve hiatal hernia and stenosis area (Figure 1).

**Figure 2:**
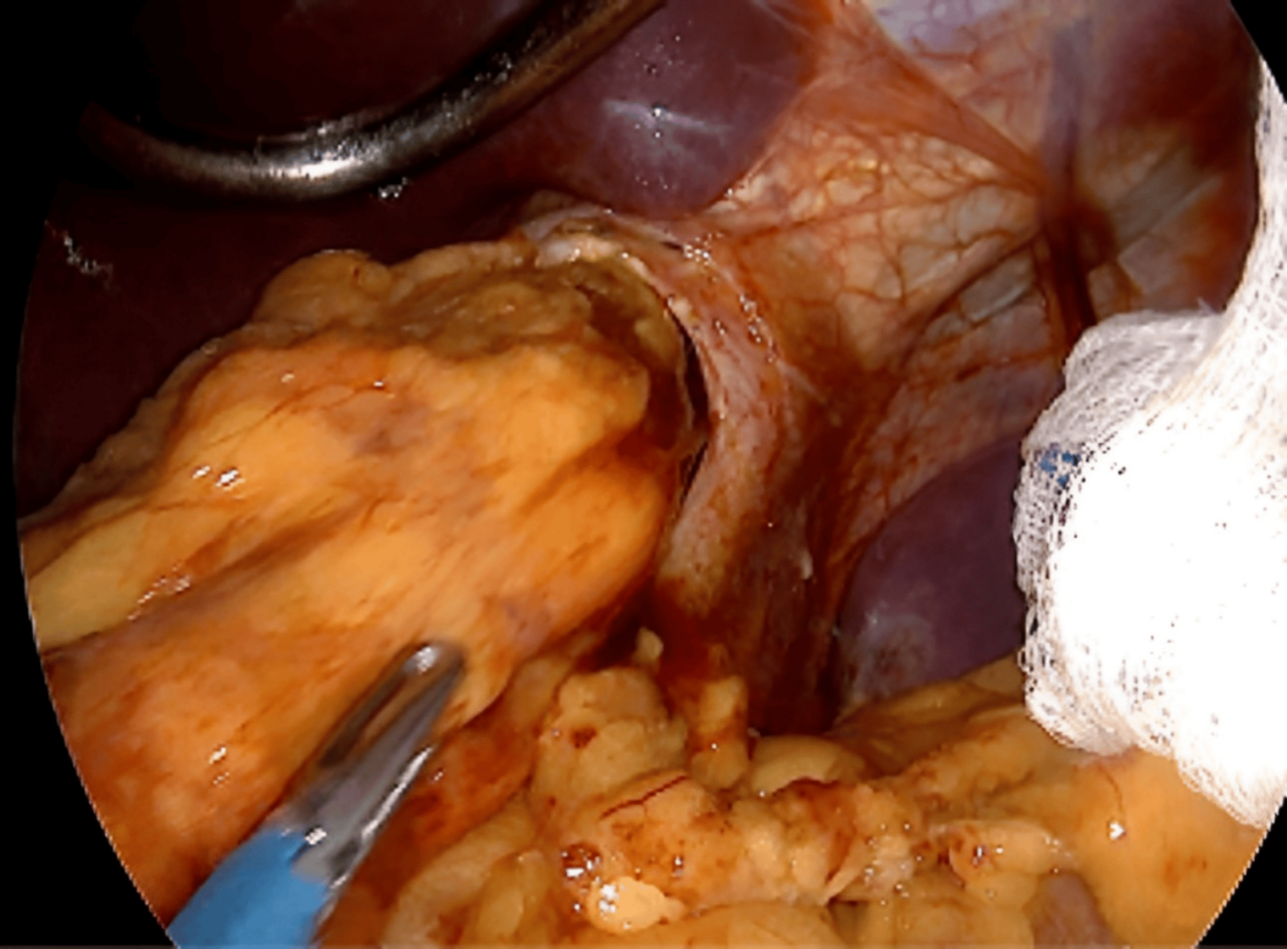
Laparoscopic view of the hernia reduction.

A diagnosis of AHH post-SG was established. The patient was scheduled for a second surgery to address the complication. During the second surgery, we found a sleeve hiatal hernia, which was managed by reducing the third proximal part of the stomach to the abdominal cavity (Figure 2).

**Figure 3:**
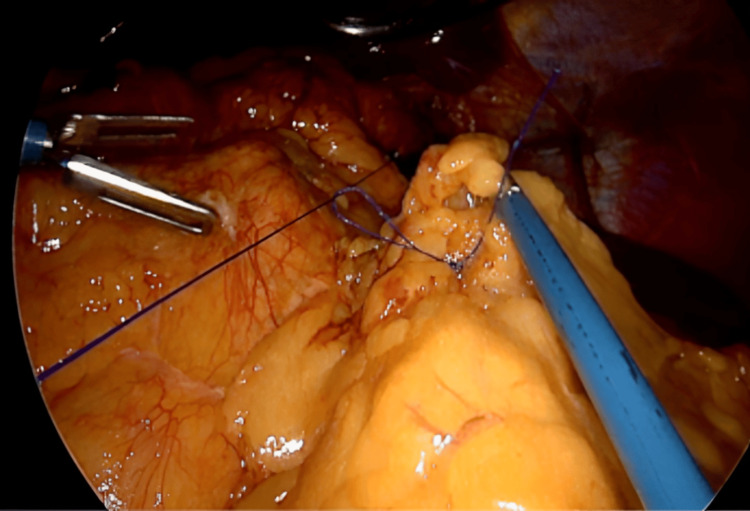
Gastric sleeve omentopexy with non-absorbable suture.

Omentopexy was performed with non-absorbable suture 2-0 (Figure 3).

The crura was not reinforced due to inflammation of the tissue. A follow-up barium swallow test conducted postoperatively showed a normal passage of barium through the esophagus and stomach, with no evidence of hiatal hernia. The patient was discharged 24 hours after the second surgery with adequate tolerance to liquids. At the one-year follow-up, the patient reported being asymptomatic with a BMI of 29.62.

## Discussion

AHH in patients who have undergone SG is a rare and underreported complication; however, if not treated promptly, it can be harmful to the patient. Very few cases have been reported in the literature, and management has not been definitively established [3-5].

In 2015, Al-Sanea et al. [3] reported a similar case in a 23-year-old male patient who presented with nausea and vomiting on his first postoperative day after SG. A diagnostic laparoscopy was performed, revealing the presence of AHH. Reduction and fixation were performed to the left anterolateral crus and the falciform ligament. This study also documents a similar case reported in 2014 in Boston by Chang, where reduction, closure of the pillars, and omentopexy were performed. Another case was reported in Israel in 2014 [4]. However, in this study, only the case was documented. It describes that the patient presented with dysphagia. The surgery is not specified, only mentioning surgical reintervention and the use of total parenteral nutrition. Pavelko reported in 2020 in the USA [5] the case of a 29-year-old female patient who presented with symptoms of vomiting and tachycardia three days after an SG procedure. A diagnostic laparoscopy was performed, and AHH was found. The management included hernia reduction and hernia closure. In our patient, it was decided to perform reduction and omentopexy (without reinforcement of the crura), which is similar to what Chang reported. Arslan et al. [8] suggested performing the omentopexy technique to reduce the incidence of AHH and gastric twists, as this technique helps stabilize the posterior gastric wall. In all the mentioned cases, reintervention was necessary, suggesting that surgical revision should be considered when suspecting this complication.

Risk factors for AHH in post-bariatric patients may include obesity, increased intra-abdominal pressure, early vomits post-surgery, dissection of the left diaphragmatic crus, and tissue laxity [3,9]. The symptoms of AHH in post-bariatric patients may include intractable vomit, oral intolerance, gastric pain, and dysphagia. Timely diagnosis can lead to interventions such as laparoscopic crura repair, fixation of the stomach to the left crus, or conversion to other bariatric procedures, as needed, to manage the hiatal hernia effectively.

In our case, the primary cause of AHH is not well established, although there are risks such as complete dissection of the phrenoesophageal membrane, dissection of the left crus, or tissue laxity in obese patients. The patient did not present preoperative symptoms or suggestive signs of hiatal hernia, highlighting the importance of considering omentopexy as a routine technique in SG to prevent its occurrence. However, further research is required [8,10].

For the diagnosis of AHH, 3D volumetry by tomography has been highlighted in several literature reports as the study with the highest diagnostic sensitivity for AHH [11,12]. However, the barium swallow test is considered by some authors as the "gold standard" approach, being a simple, cost-effective, yet highly efficient tool due to its sensitivity and rapid execution when necessary, as documented in several cases even with a relatively higher diagnostic rate than upper endoscopy [13,14].

To the best of our knowledge, this is the first reported case of AHH in our country successfully treated with the omentopexy technique. However, more studies are needed to thoroughly evaluate its effectiveness in managing this complication.

## Conclusions

AHH following SG is a rarely reported complication in the literature. The treatment has not been well established; however, if suspected, the patient should undergo surgical intervention. Hernia reduction and stomach omentopexy can be effective treatment options, but more studies are needed to confirm this approach. Each case should be individualized, with decisions based on the knowledge and experience of the surgeon and their team.
